# Results of an European survey on the management of perimesencephalic nonaneurysmal subarachnoid hemorrhage

**DOI:** 10.1038/s41598-025-06443-6

**Published:** 2025-07-08

**Authors:** Christina Wolfert, Björn Sommer, Philipp Krauss, Dorothee Mielke, Christoph J. Maurer, Ansgar Berlis, Andreas Raabe, Ehab Shiban

**Affiliations:** 1 Department of Neurosurgery, University Hospital Augsburg, Stenglinstr. 2, 86156 Augsburg, Germany; 2 Department of Neuroradiology, University Hospital Augsburg, Stenglinstr. 2, 86156 Augsburg, Germany; 3 Department of Neurosurgery, Inselspital Bern, University Hospital Bern, Rosenbühlgassse 25, Bern, 3010 Switzerland; 4 Department of Neurosurgery, Carl-Thiem Hospital Cottbus, Thiemstr. 111, 03048 Cottbus, Germany

**Keywords:** Stroke, Risk factors, Outcomes research

## Abstract

Based on the current guidelines, there is no consensus on inpatient treatment including cerebral vasospasm prophylaxis and follow-up imaging for perimesencephalic nonaneurysmal subarachnoid hemorrhage (NASAH). To evaluate the daily practice of neurosurgeons within the Vascular Section of the European Association of Neurosurgical Societies (EANS) an online survey was performed from February 2023 to June 2023. Thirty-two questionnaires were answered from eighteen different countries. Most answers were provided from employees of University Hospitals (*n* = 27; 84.4%). Up to five NASAH cases per year were reported by 10 (31.3%), another 12 (37.4%) treat more than 20 cases. The majority of contributors (65.6%) estimate the complication rates in NASAH to be less than 2%. Inpatient monitoring was significantly influenced by the initial presentation and the distribution of blood observed in the CT scan, with significantly more patients being admitted to the intensive care unit in case 3 (*p* = 0.011) compared to case 1. Further, the therapeutic approaches differ in the blood pressure monitoring (*p* = 0.08). However, this finding did not achieve statistical significance. In case 1 and case 2 neither cerebral vasospasm prophylaxis nor transcranial doppler sonography is performed in 11 centers (34.4%) which decreases statistically significantly in case 3 (*n* = 2; 6.3%; *p* = 0.0014). This study confirms that, the amount of blood in the first native CT scan influences the treatment decision. However, clear intercontinental differences cannot be evaluated due to the small number of participants.

## Introduction

Nonaneurysmal subarachnoid hemorrhage (NASAH) is defined by hemorrhage in the subarachnoid space without a detectable aneurysm or previous trauma^1^.

Depending on the specific bleeding pattern, NASAH can be categorized into perimesencephalic (PM) and non perimesencephalic (NPM) SAH^2^. According to the 2013 guidelines of the European Stroke Organization, diagnostic approaches vary based on the initial bleeding pattern. Digital subtraction angiography (DSA) remains the gold standard for the exclusion of aneurysms, however, it is only recommended in patients with NPM SAH^3^.

In contrast, due to the lower rates of rebleeding, cerebral vasospasm (CV), and delayed cerebral ischemia, these guidelines do not recommend DSA in patients suffering from PM SAH after conducting a benefit-risk analysis^3^.

Recently, a nationwide survey conducted in Germany revealed a variety of treatment approaches in these patients, being influenced by the volume of blood identified in the initial cranial computed tomography (cCT) scan^4^.

This study aims to evaluate whether this variability in treatment approaches is also prevalent across Europe.

## Materials and methods

This survey was distributed among members of the Vascular Section of the European Association of Neurosurgical Societies (EANS) and conducted via an online platform from February 2023 to June 2023.

The primary questions focused on the employment status of the respondents and details regarding patients with NASAH.

**Fig. 1:**
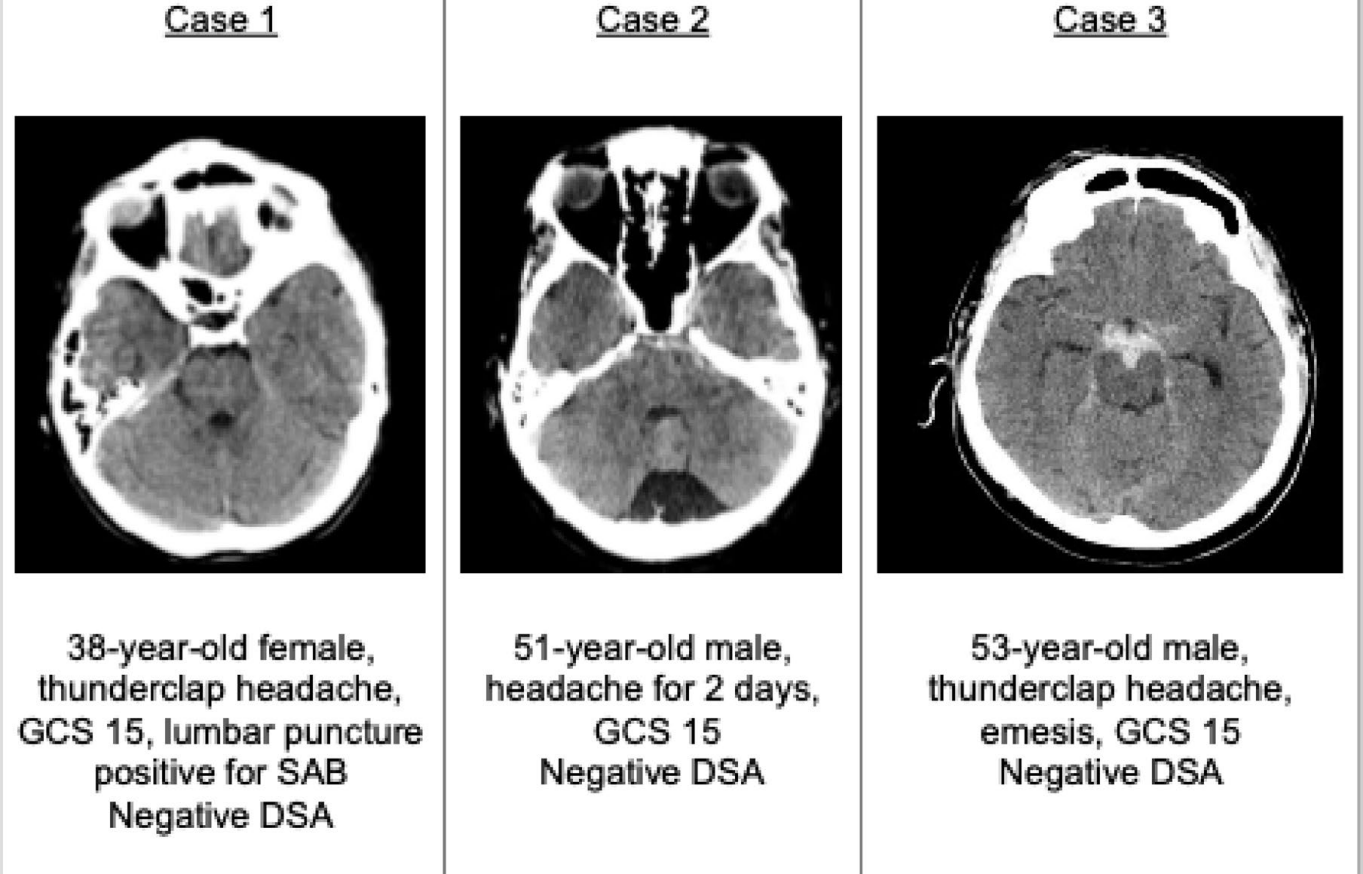
The three case vignettes.

Additionally, three clinical case vignettes were presented, illustrating varying amounts of PM SAH in the initial native cCT images. These cases are designed to facilitate the evaluation of treatment strategies and are depicted in Fig. 1.

The distributed questionnaire is available in the supplementary file.

### Aim of the study

This survey primarily focused on the caseload, the estimated complication rate, and the proposed treatment regimen of patients being admitted with NASAH to neurosurgical departments throughout hospitals of the EANS members.

The main aim was to analyze whether NASAH patients are treated differently in dependence of their clinical presentation and the amount of PM SAH detected in the initial cCT scan.

### Statistical analysis

For categorical data, absolute numbers and percentages are given. Person-Chi^2^ test without Yates correction was used to map correlation. Contingency coefficient is applied. Reported p-values are two-sided and a significance threshold of *p* < 0.05 was applied to all tests. Data analysis were performed with IBM SPSS v. 28 (IBM Corp, Armonk, USA).

### Ethical approval and consent to participate

This multicentric survey was carried out in accordance with the ethical standards of the institutional and national research committee and with the 1964 Helsinki Declaration and later amendments. The Human Investigation Committee of the Ludwig-Maximilians-University Munich, Germany approved the survey (Reference Nr: 21–1297). Individual consent to participate was not necessary, since the survey was conducted without real information about the individual patients.

## Results

### Response rates

**Fig. 2:**
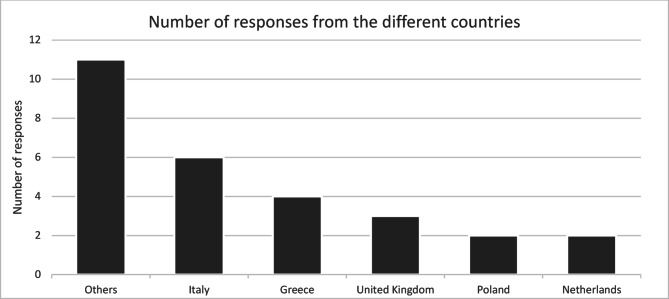
Response rates of the respective countries.

Thirty-two questionnaires were returned from eighteen different countries. Most were completed by employees from departments located in Italy (*n* = 6; 18.8%), Greece (*n* = 4; 12.5%), or the United Kingdom (*n* = 3; 9.4%). Questionnaires from countries with only one response were grouped under the heading “Others”. Details are shown in Fig. 2.

### Employment status

Four (12.5%) of the respondents were employed as neurosurgical residents. Twice as many (*n* = 8; 25.0%) were consultants or head of the departments. The remainder (*n* = 12; 37.5%) were employed as senior consultants.

Most of the responding centers were university hospitals (*n* = 27; 84.3%). The remaining five were maximum care hospitals (*n* = 3; 9.4%), or private clinics (*n* = 2; 6.3%).

### Estimated caseload

**Fig. 3:**
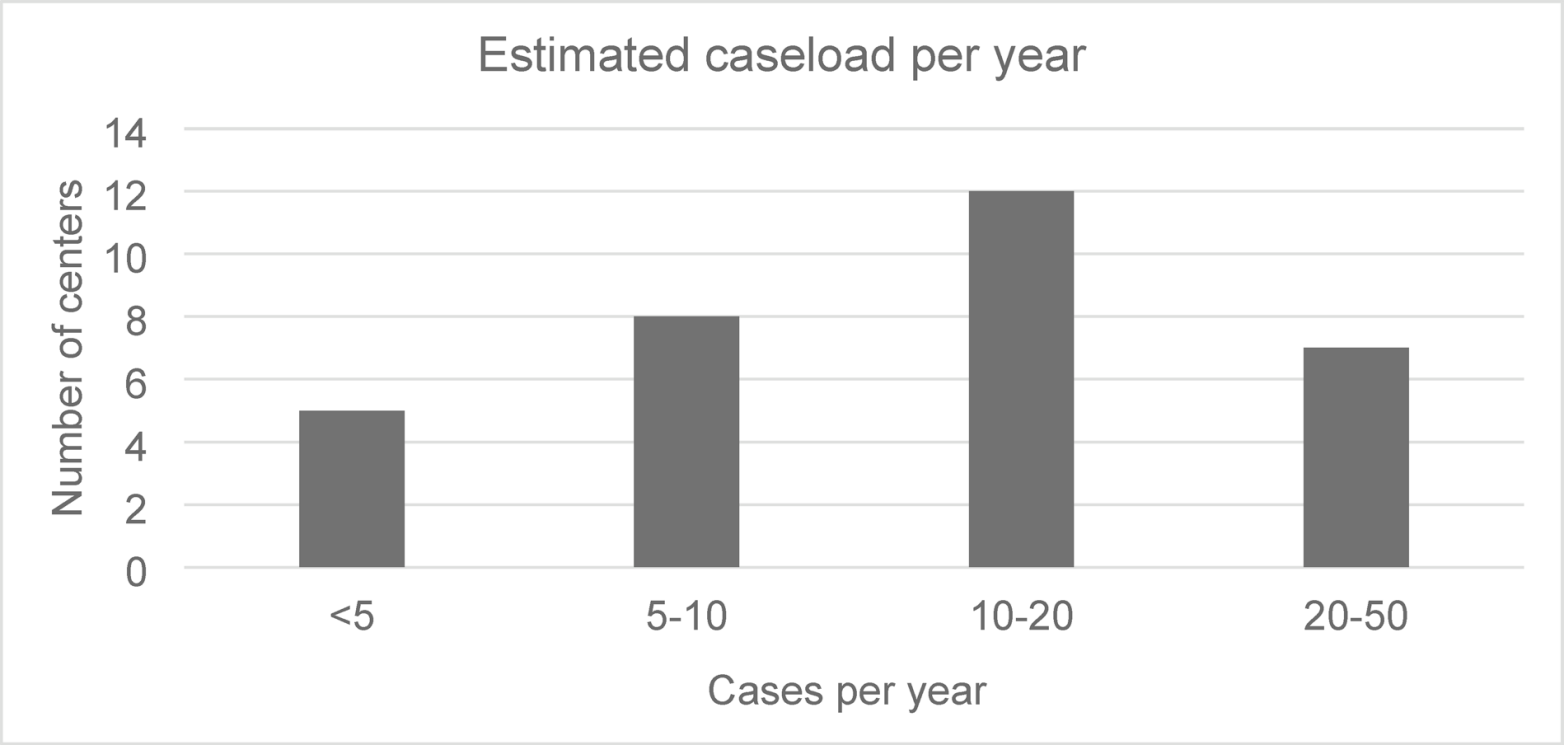
Annual caseload of NASAH patients per center.

More than one third of the centers (*n* = 12; 37.5%) treat 10–20 NASAH patients per year, while a caseload of less than five cases annually is rare (*n* = 5; 15.6%). Detailed information is presented in Fig. 3.

### Complication rate

Three centers (9.3%) report no complications in these patients. In addition, most contributors (*n* = 18; 56.3%) estimate the complication rate to be less than 2%. While complication rates exceeding 10% are stated by *n* = 3 (9.3%). No notable correlation was identified in the estimated complication rate and the hospital’s care status.

**Fig. 4:**
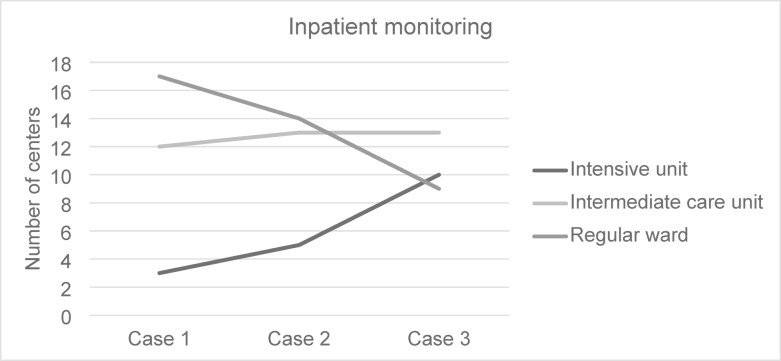
Inpatient monitoring after admission.

### Inpatient monitoring

In the first case, 3 centers (9.3%) admit their patient to the intensive care unit (ICU), 12 (37.5%) to the intermediate care unit (IMCU), and 17 (53.1%) to the regular ward (RW). However, the management changes with increasing amount of PM SAH detected on the cCT scan. Comparing the answers for inpatient monitoring given for case 1 and case 3, statistical significance was reached comparing monitoring on the RW and ICU (*p* = 0.017; *p* = 0.011). Inpatient monitoring is shown in Fig. 4.

### Blood pressure monitoring

**Fig. 5:**
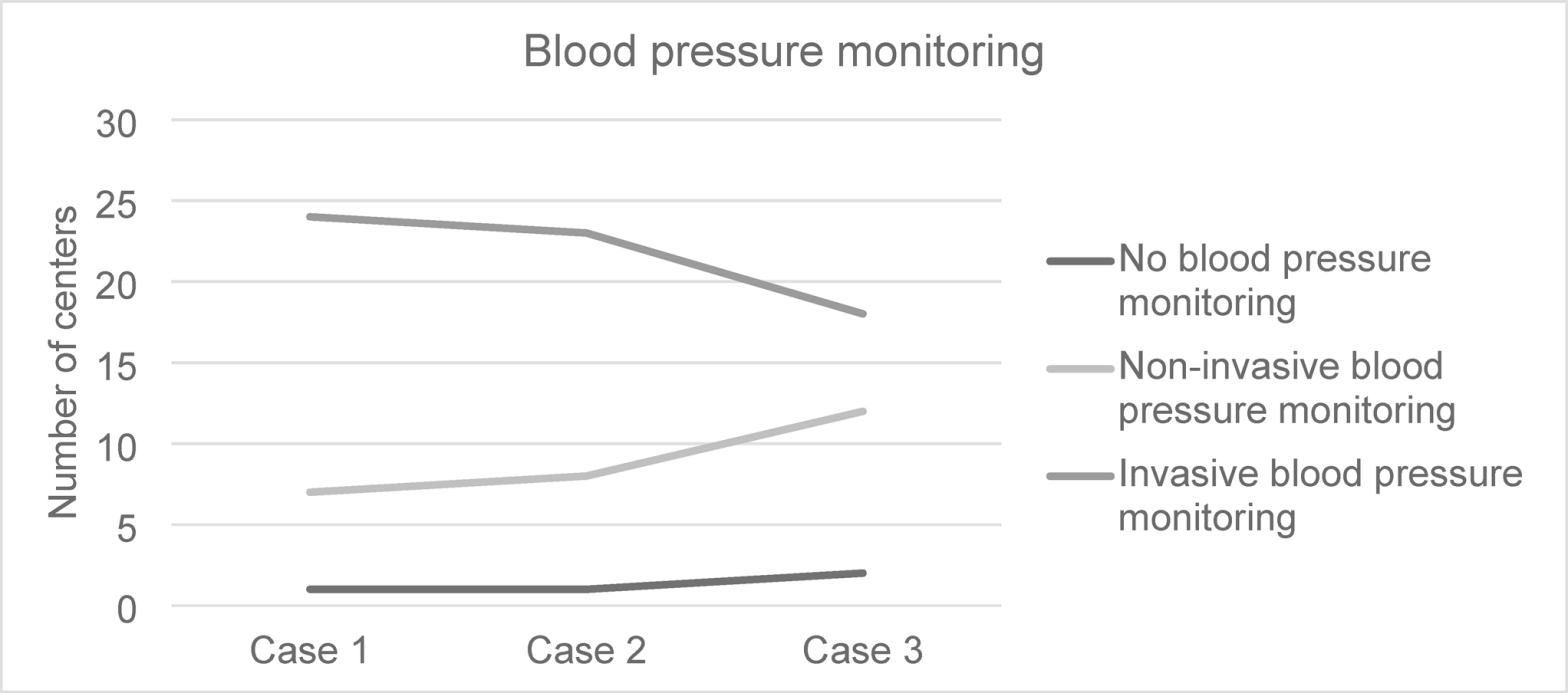
Blood pressure monitoring in the different cases.

One center did not use any blood pressure monitoring in all cases (3.1%), and another did not use it in case 3 (3.1%). The method used for blood pressure measurement was variable, with elevated numbers of invasive monitoring being used in case 3. Nonetheless, these results did not reach statistical significance (*p* = 0.08). Details are shown in Fig. 5.

### Treatment regimens

**Fig. 6:**
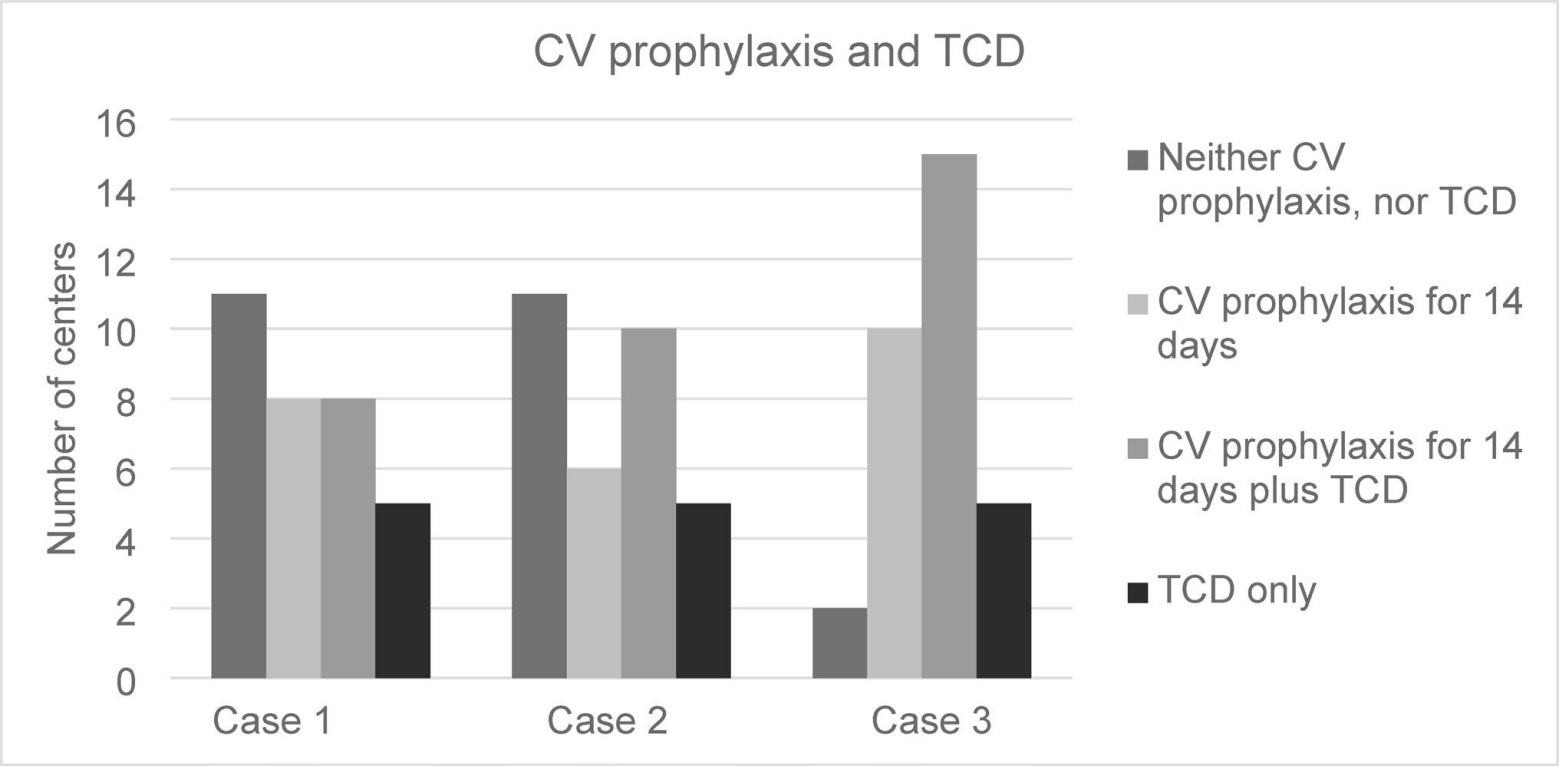
Treatment regimens with/without CV prophylaxis and TCD.

Comparing all treatment regimens, the number of patients in the group “neither CV prophylaxis nor transcranial doppler sonography (TCD)” was statistically significantly reduced in case 3 (*p* = 0.0014). Details Fig. 6.

### Follow-up imaging

Statistically significantly more centers perform a second DSA in case 3 compared to case 1 and case 2 (*p* = 0.027; *p* = 0.014). This DSA is most often scheduled during the hospital stay or within the first three months after the initial bleeding. Baseline data is summarized in Table 1.

**Table 1:** Timing and performance of follow up DSA.

	Case 1 (*n*; %)	Case 2 (*n*; %)	Case 3 (*n*; %)	*p*-value Case 1: Case 2	*p*-value Case 1: Case 3	*p*-value Case 2: Case 3
**Treatment regimen**						
RW	17 (53.1)	14 (43.8)	9 (28.1)	0.77	0.017*	0.90
MICU	12 (37.5)	13 (40.6)	13 (40.6)	0.39	0.39	0.50
ICU	3 (9.4)	5 (16.6)	10 (31.3)	0.22	0.011*	0.06
**BP Monitoring**						
Invasive	7 (21.9)	8 (25.0)	12 (37.5)	0.38	0.08	0.13
Non-invasive	24 (75.0)	23 (71.9)	18 (56.3)	0.61	0.94	0.90
No BP Monitoring	1 (3.1)	1 (3.1)	2 (6.3)	0.50	0.27	0.27
**CV prophylaxis**						
CV prophylaxis + TCD	8 (25.0)	10 (31.3)	15 (46.8)	0.28	0.96	0.09
CV prophylaxis without TCD	8 (25.0)	6 (18.8)	10 (31.3)	0.72	0.71	0.12
TCD without CV prophylaxis	5 (16.6)	5 (16.6)	5 (16.6)	0.50	0.50	0.50
Neither CV nor TCD prophylaxis	11 (34.4)	11 (34.4)	2 (6.3)	0.50	0.0014*	0.0014*
**Second DSA**						
No 2nd DSA	14 (43.8)	15 (46.8)	7 (21.9)	0.59	0.027*	0.014*
During the hospital stay	9 (28.1)	8 (25.0)	15 (46.8)	0.59	0.94	0.28
After 6–12 weeks	8 (25.0)	8 (25.0)	8 (25.0)	0.50	0.50	0.50
After 6–12 month	1 (3.1)	1 (3.1)	3 (9.4)	0.50	0.50	0.14
**Third DSA**						
3rd DSA	3 (9.4)	2 (6.3)	5 (16.6)	0.67	0.22	0.11
No 3rd DSA	29 (90.6)	30 (93.7)	5 (16.6)	0.32	0.22	0.88

## Discussion

The current study presents the findings of our survey, encompassing diagnostic and therapeutic strategies in patients with NASAH - initially confirmed by one negative DSA - within neurosurgical departments of the EANS. Our results indicate statistically significant variations in the treatment protocols for inpatient monitoring, specifically regarding CV prophylaxis and TCD, as well as the execution of a follow-up DSA - based on the amount of PM SAH detected in the first native cCT.

NASAH is frequently classified as a “benign” form of bleeding, attributed to its lower complication rates^5,6^. Therefore, inpatient treatment including CV prophylaxis and repetitive TCD are rarely evaluated^3^. The lower complication rate is supported by many studies, exemplary a work conducted by Alrohimi and colleagues in 2024 compared aneurysmal SAH with NASAH, revealing a statistically significant disparity in the incidence of symptomatic vasospasm (40% vs. 2.6%; *p* = 0.01)^6^. This finding aligns with the estimated complication rate (> 5%), which were reported by 84.4% of our respondents. The estimated complication rates did not differ regarding the employment status of the respondents. These results are consistent with the results of our nationwide survey in Germany, with a complication rate minor 5% being reported from 76.3% respondents^4^.

Currently, DSA is not recommended for these patients based on a benefit-risk analysis^3^. Nevertheless, numerous neurosurgical departments continue to perform at least one initial DSA, and more and more data is being published regarding necessity and timing of follow-up DSA^4,7,8^. This reflects a cautious stance among clinicians, despite a recent study by Vogentseder and colleagues excluding any bleeding source in 51 patients with PM SAH^9^. Our study was able to confirm this cautious approach of neurosurgeons within the Vascular Section of the EANS, as evidenced by a statistically significant increase in the performance of a secondary DSA in case 3 compared to cases 1 and 2.

In relation to inpatient care, a statistically significant increase in the number of patients being admitted to the ICU in case 3 was observed when compared to the other cases (*p* = 0.011). Although this clinical pathway is not recommended, the already published survey conducted in Germany and this survey including experts of the Vascular Section of the EANS, revealed that clinicians adapt their daily practice to the individual needs and presentations of patients^4^. This finding underscores the variability in treatment protocols across different institutions and emphasizes the necessity for standardized guidelines tailored to the specific characteristics of NASAH.

In conclusion, while NASAH typically presents a less severe clinical profile compared to aneurysmal SAH, its diagnosis and management require a careful consideration of bleeding patterns and individual patient factors, including the risk of secondary aneurysm finding. Based on our hospital guidelines, we recommend an initial DSA to rule out vascular pathologies. In case of cerebral vasospasm, a high Fisher grade and/or a high World Federation of Neurosurgical Societies (WFNS) grade, a second DSA within the hospital stay should be performed. Furthermore, ongoing research and consensus building among healthcare providers are essential to establish effective treatment protocols and optimize patient outcomes.

### Strengths and limitations

This survey was conducted within the vascular section of the EANS. Although centers from eighteen different countries participated, the response rate does not permit any significant conclusions regarding the geographical distribution of NASAH and its associated treatments. Furthermore, the number of NASAH patients treated annually and the complication rates are only estimated numbers which are not objectively assessed. As this survey exclusively focuses on DSA as a diagnostic tool, no inferences can be made concerning additional diagnostic modalities such as CT or MRI with contrast agents.

## Data Availability

Availability of materials and data. Data is available upon reasonable request. Request should be addressed to C.W.
